# Genomic DNA Copy-Number Alterations of the *let-7* Family in Human Cancers

**DOI:** 10.1371/journal.pone.0044399

**Published:** 2012-09-06

**Authors:** Yanling Wang, Xiaowen Hu, Joel Greshock, Liang Shen, Xiaojun Yang, Zhongjun Shao, Shun Liang, Janos L. Tanyi, Anil K. Sood, Lin Zhang

**Affiliations:** 1 Department of Obstetrics and Gynecology; University of Pennsylvania, Philadelphia, Pennsylvania, United States of America; 2 Abramson Family Cancer Research Institute; University of Pennsylvania, Philadelphia, Pennsylvania, United States of America; 3 Department of Gynecologic Oncology, Division of Surgery, University of Texas MD Anderson Cancer Center, Houston, Texas, United States of America; Florida International University, United States of America

## Abstract

In human cancer, expression of the *let-7* family is significantly reduced, and this is associated with shorter survival times in patients. However, the mechanisms leading to *let-7* downregulation in cancer are still largely unclear. Since an alteration in copy-number is one of the causes of gene deregulation in cancer, we examined copy number alterations of the *let-7* family in 2,969 cancer specimens from a high-resolution SNP array dataset. We found that there was a reduction in the copy number of *let-7* genes in a cancer-type specific manner. Importantly, focal deletion of four *let-7* family members was found in three cancer types: medulloblastoma (*let-7a-2* and *let-7e*), breast cancer (*let-7a-2*), and ovarian cancer (*let-7a-3*/*let-7b*). For example, the genomic locus harboring *let-7a-3*/*let-7b* was deleted in 44% of the specimens from ovarian cancer patients. We also found a positive correlation between the copy number of *let-7b* and mature *let-7b* expression in ovarian cancer. Finally, we showed that restoration of *let-7b* expression dramatically reduced ovarian tumor growth *in vitro* and *in vivo*. Our results indicate that copy number deletion is an important mechanism leading to the downregulation of expression of specific *let-7* family members in medulloblastoma, breast, and ovarian cancers. Restoration of *let-7* expression in tumor cells could provide a novel therapeutic strategy for the treatment of cancer.

## Introduction

The characterization of heterochronic mutations in *Caenorhabditis elegans* revealed an evolutionarily conserved genetic pathway that orchestrates both the timing of cell divisions and cellular fates, as appropriate for the developmental stage of the organism [1]–[5]. In this pathway, the microRNA (miRNA) *let-7* controls the progression of timing events, ensuring that cell cycle exit and terminal differentiation occur at the correct time [6], [7]. The *let-7* target genes *lin-28* (an RNA-binding protein) and *lin-41* (a putative ubiquitin ligase) block *let-7* maturation and interact with argonaute proteins, respectively. These heterochronic genes appear to form bistable switches via double-negative regulatory feedback loops [2]–[5]. Expression of *LIN28* and *LIN41* is highly restricted at undifferentiated stages, such as in embryonic stem cells, early embryos, and certain somatic stem/progenitor cells. Conversely, their regulatory miRNA, *let-7*, shows a reciprocal temporal expression pattern that is dramatically increased during differentiation and development, and it is extensively expressed in adult tissues [2]–[5]. Thirteen members of the *let-7* family have been identified in the human genome [7], [8] which display both distinct and overlapping functions [8].

The role of *let-7* in cancer was first discovered by Johnson *et al.* when they found that the *let-7* family negatively regulates *let-60/RAS* in *C. elegans* by binding to multiple *let-7* complementary sites in its 3′ untranslated region (3′UTR) [9]. Moreover, having found that *let-7* expression is lower in lung tumors than in normal lung tissue, while *RAS* protein is significantly higher in lung tumors, they proposed that *let-7* is a tumor suppressor gene [9], which is consistent with previous clinical observations in lung cancer [10]. Reduced expression of *let-7* has been associated with shortened postoperative survival in patients with cancer [7], [11], [12], and forced expression of *let-7* family members can suppress cancer cell growth both *in vitro* and *in vivo* [13]–[16]. The inhibitory function of the *let-7* family in cancer has been corroborated by a number of groups and in various types of tumors [7], [11], [12]. It is likely that *let-7* performs these functions by targeting various genes. For example, *let-7* inhibits many well-characterized oncogenic proteins, including *KRAS* [9], [17], [18], *HRAS* [9], [17], [18], *HMGA2* [18]–[21], *c-Myc* [22], and *NF2* [23]. In addition, *let-7* targets multiple cell cycle associated genes, including *CDC25A* [24], *CDK6* [24], and *CDK4* [25] as well as *Cyclin A* [25], *D1* [25], *D2* [24], and *D3* [25]. Finally, *let-7* represses expression of the reprogramming factor *LIN28* that functions to block differentiation and maintain cancer stem cell populations [26].

Since it has been found that human cancers show a significantly reduced expression of the *let-7* family, and that this is associated with shorter survival times in these patients [7], [11], [12], the characterization of the mechanisms leading to *let-7* downregulation in cancer has important clinical significance. One cause of genetic deregulation in cancer is an alteration in the somatic copy-number of a given gene [27]. It has also been reported that miRNA copy numbers may be altered during tumorigenesis [28], [29]. This led us to examine whether the copy-numbers of the *let-7* family were altered in cancer.

## Results

### Certain Members of the *let-7* Family have Deletions in Copy Number in Medulloblastoma, Breast Cancer, and Ovarian Cancer

**Figure 1:**
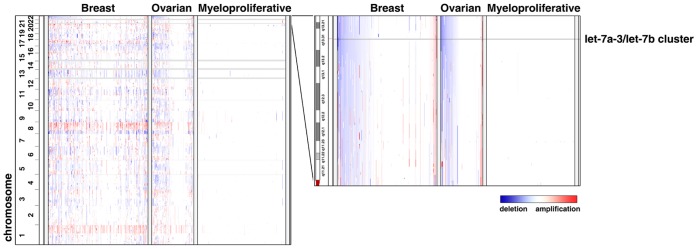
The genomic locus harboring the *let-7a-3*/*let-7b* cluster shows copy number deletions in breast and ovarian cancers. The segmented raw data from the SNP arrays (breast cancer, n = 293; ovarian cancer, n = 110; myeloproliferative disorder, n = 215) was retrieved from the Tumorscape database, analyzed, then visualized by the Integrative Genomics Viewer. Left panel: whole genome wide view of the copy number profiles from myeloproliferative disorder, breast cancer, and ovarian cancer (genomic locations are on the left; tumor specimens are across the top). Red represents amplification and blue represents deletion. Right panel: Copy-number profiles of chromosome 22 in the region of the *let-7a-3*/*let-7b* cluster.

To determine the copy number of the *let-7* family members, we analyzed a high-resolution SNP array (Affymetrix 250 K Sty array) dataset, Tumorscape, created by the Broad Institute of MIT and Harvard [27]. Fourteen types of human cancers (a total of 2,969 tumor specimens) were included in our study (Table 1). The segmented raw data of the above SNP arrays were retrieved and analyzed following a standard protocol provided by the Tumorscape database [27] and visualized by the Integrative Genomics Viewer (IGV) (Figure 1) [30]. The genomic regions where the copy number alterations occurred significantly more frequently than the background rate were identified using GISTIC algorithm [31]. The following terms, which were defined by the Tumorscape database [27], were used to describe our results. Frequency indicates the fraction of cancers which exhibit amplification/deletion at the genomic locus harboring a given *let-7* gene. Low q-values (upper threshold = 0.25) suggest that amplifications/deletions at this locus are significant and enriched by selective pressures.

**Table 1:** Summary of the samples from the Tumorscape database used in this study.

Tumor Type	Number of specimens	Number of cell lines	Total number of samples
**Ovarian**	103	7	110
**Breast**	243	50	293
**Lung SC**	40	23	63
**Lung NSC**	733	105	838
**Colorectal**	161	33	194
**Prostate**	92	9	101
**Esophageal squamous**	44	12	56
**Renal**	126	27	153
**Hepatocellular**	121	11	132
**Melanoma**	111	108	219
**Acute lymphoblastic leukemia**	391	13	404
**Myeloproliferative disorder**	215	0	215
**Glioma**	41	13	54
**Medulloblastoma**	128	9	137
**Total**	**2549**	**420**	**2969**

**Figure 2:**
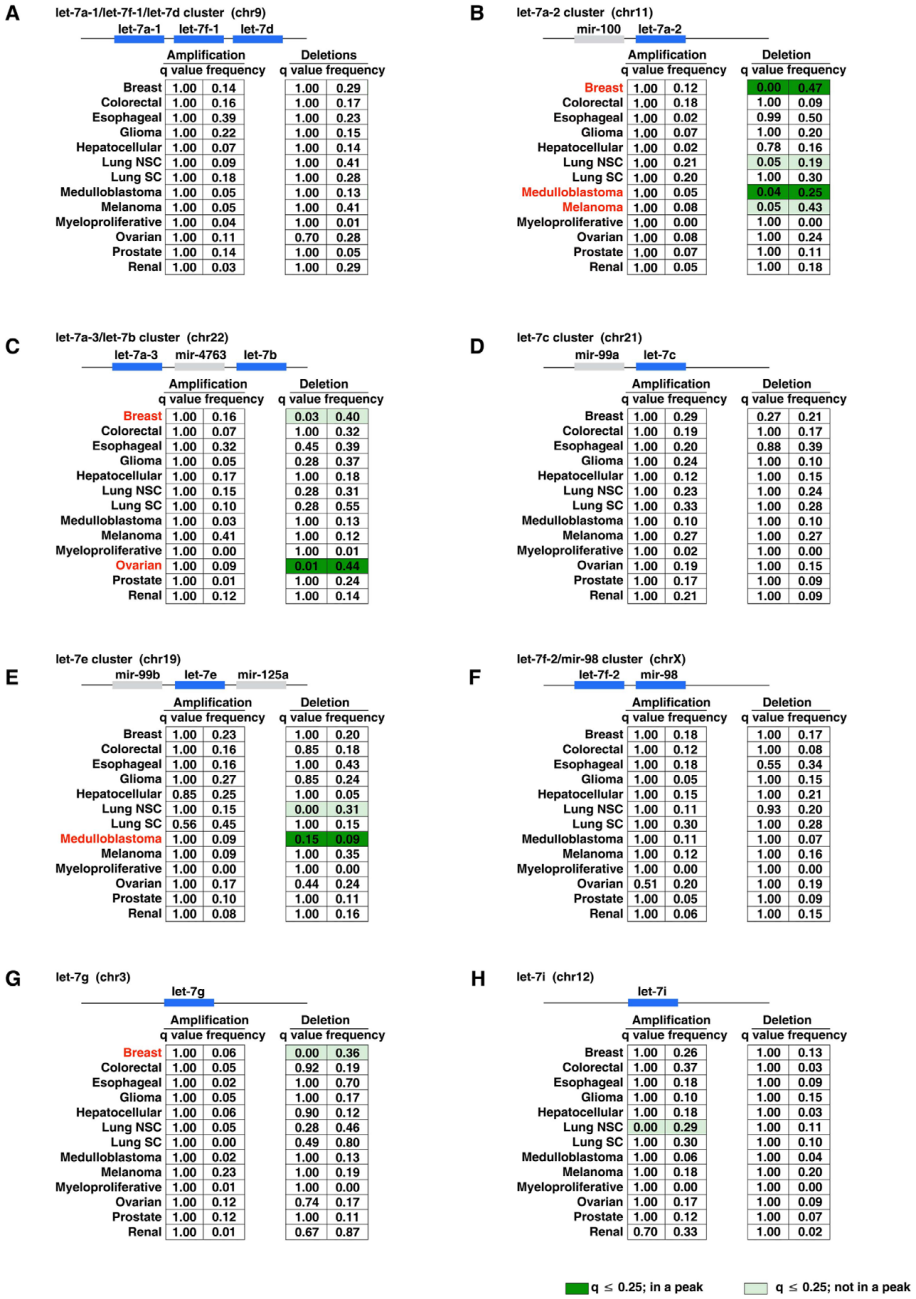
Members of the *let-7* family show copy number deletions in medulloblastoma, breast, and ovarian cancers. Summary of DNA copy number alterations of the *let-7* family in 14 types of human cancers (n = 2,969). The *let-7* family is made up of thirteen members located at eight loci of the human genome. Many of them, such as *let-7a-1*, *let-7f-1* and *let-7d*, cluster together (A). Low q-values (upper threshold = 0.25) suggest that amplifications/deletions at this locus are significant and enriched by selective pressures. Dark green represents focal deletion of the *let-7* family.

As shown in Figure 2, the *let-7* family is present as thirteen members located at eight loci in the human genome [7], [11], [12]. All eight genomic loci were analyzed in our study. Most of the family members, such as *let-7a-1*, *let-7f-1,* and *let-7d*, cluster together. (The *let-7* genes are named by including a letter to denote a distinct mature sequence, and a number to indicate that the same mature sequence is present at distinct genomic loci.) Briefly, we found that four *let-7* loci harboring five *let-7* members showed significant deletions in copy number in a cancer-type specific manner (Figure 2). Focal deletions of these *let-7* family members were found in three cancer types: medulloblastoma (*let-7a-2,* frequency 25%; *let-7e,* frequency 9%), breast cancer (*let-7a-2,* frequency 47%), and ovarian cancer (*let-7a-3*/*let-7b,* frequency 44%). This suggests that genomic focal copy number deletions of *let-7* may play an important role during tumorigenesis in the above cancer types. In addition, two non-focal deletions (*let-7a-3*/*let-7b,* frequency 40%; *let-7*g, frequency 36%) were also found in breast cancer, and single non-focal deletions were found in melanoma (*let-7a-2,* frequency 43%) and non-small cell lung carcinoma (*let-7e,* frequency 31%). Finally, only one *let-7* family member, *let-7i*, was found to be amplified (in non-small cell lung carcinoma). However, this was appeared a chromosome arm-level, non-focal amplification, indicating that it could be a “passenger” genetic alteration. Taken together, our data indicate that a reduction in copy number of specific *let-7* family member genes were frequent in medulloblastoma, breast, and ovarian cancers.

### Copy Number Alteration of *let-7b* is Positively Correlated with Mature *let-7b* Expression in Ovarian Cancer

**Figure 3:**
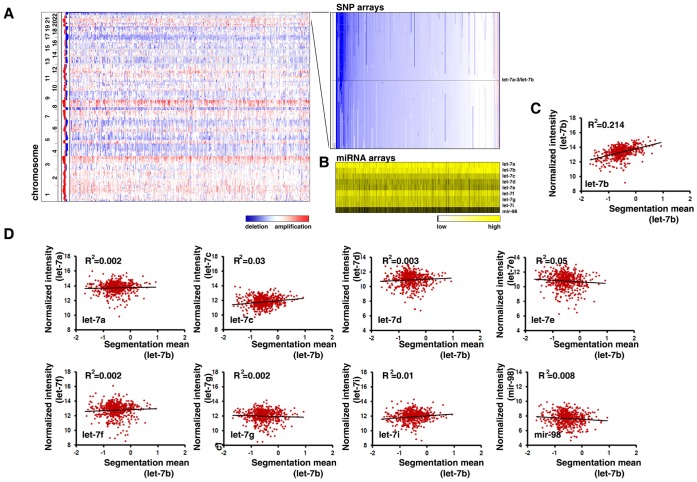
Copy number alteration of *let-7b* is positively correlated with mature *let-7b* expression in ovarian cancer. Level 3 data (segmented SNP array data and normalized miRNA array data) was retrieved from TCGA. A total of 537 tumors with both SNP and miRNA arrays data were identified. **A.** Left panel: whole genome wide view of copy number profiles in ovarian cancer (genomic locations are on the left; tumor specimens are across the top). Red represents amplification and blue represents deletion. Right panel: Copy-number profiles from chromosome 22 in the region of the *let-7a-3*/*let-7b* cluster. **B.** Heat map of mature *let-7* family expression levels in matched TCGA specimens. The samples are arranged in the same order as the SNP data. **C.** Correlations between *let-7b* DNA copy number and mature *let-7b* expression levels in ovarian cancer from the TCGA dataset. **D.** Correlations between *let-7b* DNA copy number and expression levels of mature miRNA of other *let-7* family members in ovarian cancer from the TCGA dataset.

To determine whether copy number alterations of *let-7b* affect mature *let-7* expression in cancer, we examined an ovarian cancer dataset from The Cancer Genome Atlas (TCGA) [32], because this independent genomic dataset contains matched data on both genome-wide copy number (SNP array) and mature miRNA expression (miRNA array) from a large collection of human ovarian tumor specimens. Level 3 data (segmented SNP array data and normalized miRNA array data) was retrieved from TCGA [32]. A total of 537 tumors with data from both the SNP and miRNA arrays were identified. Consistent with the data from our Tumorscape analysis, there was a deletion in copy number at the *let-7a-3*/*let-7b* locus in the specimens from ovarian cancer patients (Figure 3A). Since the mature *let-7*a sequence is encoded by three *let-7*a genes, which are located at three different chromosomal loci, we could not examine the correlation between copy number and expression for *let-7a-3*. Thus, we analyzed the correlation between deletions at the *let-7a-3*/*let-7b* locus and expression of mature *let-7b* in this data set. As shown in Figure 3 B and C, the expression of mature *let-7b* was significantly and positively correlated with *let-7b* copy number in ovarian cancer specimens (p<0.0001, R = 0.46, n = 537). We did not find a correlation between any other *let-7* family members with the *let-7b* copy number (Figure 3D). This demonstrates that a reduction in the copy number of *let-7b* leads to a reduction in the expression of mature *let-7b* in ovarian cancer.

### Restoration of *let-7b* Expression Significantly Reduces Ovarian Tumor Growth *in vitro*

**Figure 4:**
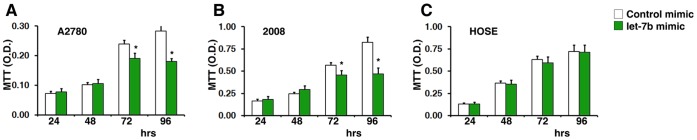
Restoration of *let-7b* expression significantly reduces ovarian tumor growth *in vitro*. The *let-7b* mimic and control oligo (30 nM) were transfected into the A2780 (A), 2008 (B) and HOSE (C) cells by lipofectamine. The cell growth was monitored by a MTT assay.

Focal loss in copy number of the*let-7* family members in medulloblastoma, breast, and ovarian cancers strongly suggests that *let-7* may have an important role in tumorigenesis. Therefore, restoring its expression could suppress tumor growth in these cancers. To test this hypothesis *in vitro,* we transfected two ovarian cancer cell lines, A2780 and 2008, with a *let-7b* mimic (a double-strand RNA oligonucleotide). A cultured human ovarian surface epithelial (HOSE) cell line was used as a control, and cell growth was monitored via an MTT assay. At 72 hours post-transfection, we found that the *let-7b* mimic significantly reduced cell growth in the cancer cells (Figure 3 A and B). There was no reduction in cell growth seen in the control cells (Figure 4C). This suggests that the restoration of *let-7b* expression in tumor cells may have great therapeutic potential for the treatment of ovarian cancer.

### Restoration of *let-7b* Expression Significantly Reduces Ovarian Tumor Growth *in vivo*

**Figure 5:**
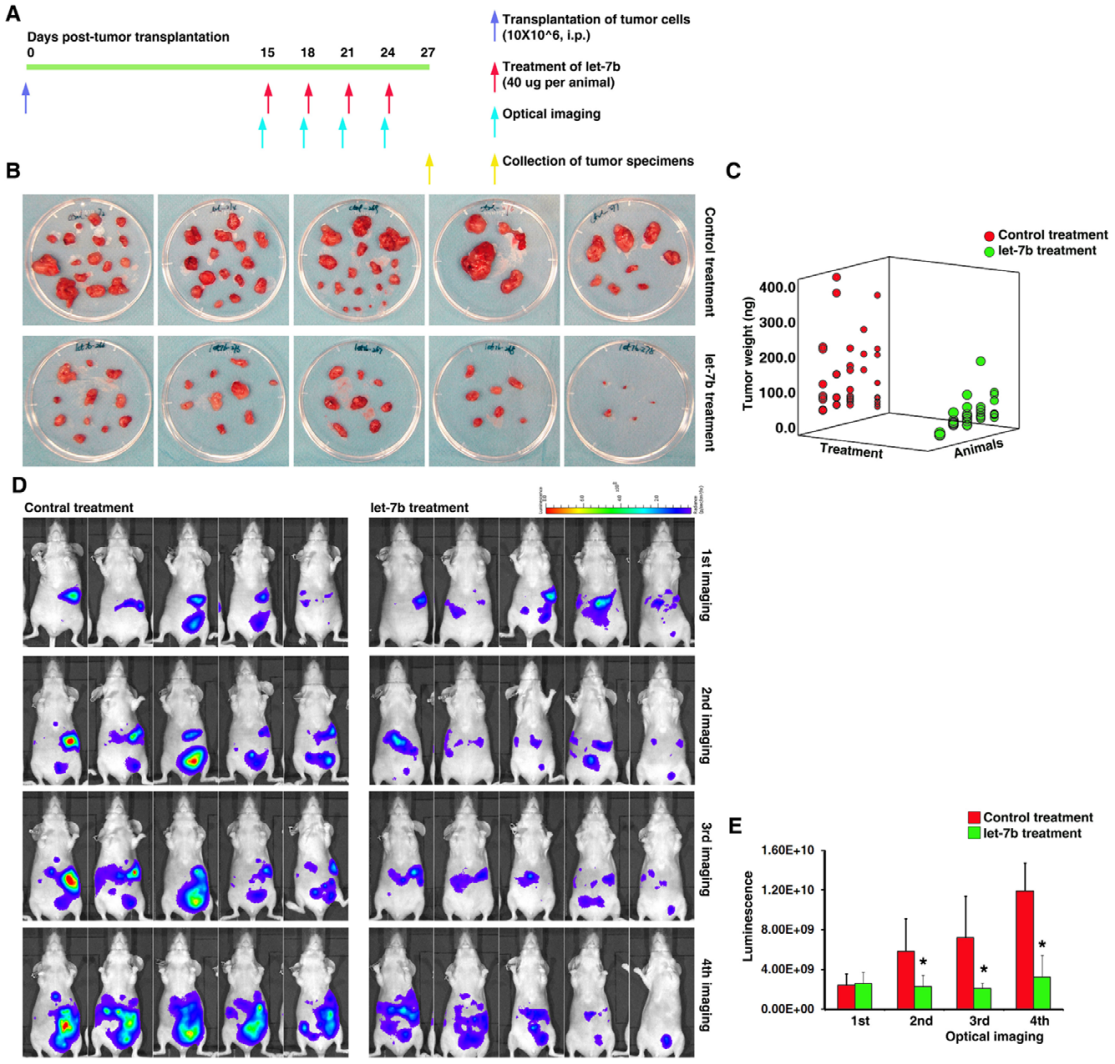
Restoration of *let-7b* expression significantly reduces ovarian tumor growth *in vivo.* A. Timeline of transplantation of tumor cells by intraperitoneal injection, treatment, imaging, and collection of tumor samples in the orthotopic late stage ovarian cancer mouse model. **B.** Tumor nodes collected from each mouse three days after the last treatment. **C.** Summary of the weights of the tumor nodes from each mouse. **D.** Endogenous *let-7b* activity in each mouse as monitored by a *let-7b* luciferase sensor. **E.** Summary of the luciferase intensity during the treatment.

To evaluate the *in vivo* therapeutic potential of the *let-7b* mimic, an orthotopic late stage ovarian cancer mouse model was generated by intraperitoneal injection of 10×10^6^ ovarian cancer (A2780) cells into female nude mice (Figure 5A). After two weeks, the mice were randomly assigned to two groups, to be treated with either the *let-7* mimic or the control oligonucleotide by i.p. injection. The mice were treated every three days for a total of four treatments (Figure 5A), and the tumor nodes were collected three days after the last treatment (Figure 5B). We found that the weight of the tumor nodes was significantly reduced in the mice treated with the *let-7b* mimic compared to controls (Figure 5 B and C). We also monitored endogenous *let-7b* activity using a constitutively expressed *let-7b* luciferase reporter that contained sequences complementary to *let-7* in the 3'UTR [33], [34]. We consistently found that treatment with the *let-7b* mimic significantly decreased luciferase activity *in vivo* compared to the control group (Figure 5 D and E). Finally, we found that treatment with the *let-7b* mimic significantly increased the *let-7b* expression levels in the treatment compared to the control group (7.03±5.73 fold, p = 0.047). Meanwhile, the mRNA expression levels of well-known *let-7* target genes such as *CCND1*, *CDC25A*, *HMGA2*, *IL6* and *LIN28B* were significantly decreased by *let-7b* mimic treatment. Taken together, this demonstrates that the *in vivo* delivery of a *let-7b* mimic can functionally restore *let-7* expression and remarkably reduce tumor growth in a pre-clinical animal model of ovarian cancer.

## Discussion

The *let-7* family is one of the first miRNA tumor suppressor families shown to be involved in human cancer. Expression of members of the *let-7* family has been reported to be significantly downregulated in multiple cancer types, and this decreased *let-7* expression has been correlated with poorer clinical outcomes. Two molecular mechanisms have been proposed that may lead to global downregulation of *let-7* expression in cancer. First, key proteins in the miRNA biogenesis pathway, such as Dicer and Drosha, can be remarkably deregulated in cancer [35]. This may result in an unselective, global downregulation of miRNAs, including the let-7 family. In addition, it has been shown that the RNA-binding protein, *LIN28,* which selectively inhibits some miRNA families, including the *let-7* family [36]–[41], is activated in a large percentage of cancer patients [42]–[51]. However, the above two mechanisms cannot explain the finding that, in most cancer types, only some *let-7* family members are downregulated. For example, the expression of *let-7*a, *let-7*c, and *let-7*g have been found to be selectively downregulated in breast cancer [52], suggesting that there are other independent mechanisms affecting the expression of each individual *let-7* family member.

Recent advances in high-throughput genome characterization technologies have revealed that cancer genomes are highly disordered, with extensive changes in chromosome structure and gene copy numbers. It has also been reported that copy numbers of miRNAs are commonly altered in human cancer [28], [29]. For example, the *mir-16-1/mir-15a* cluster on chromosome 13q14 was deleted in more than 50% of the chronic lymphocytic leukemia patients [53]. In addition, amplification of the *C13orf25/mir-17∼92* cluster on chromosome 13q31-32 has been reported in lymphoma patients [54], [55]. In the present study, we have shown that three *let-7* loci, which harbor four *let-7* members (*let-7a-2*, *let-7a-3*, *let-7b*, and *let-7e*), have deletions in copy number in a cancer-type specific manner in medulloblastoma, breast cancer, and ovarian cancer. Most importantly, we confirmed the correlation between *let-7* copy number alterations and mature *let-7* expression in ovarian cancer. These results indicate that deletion in copy number is an important mechanism leading to the downregulation of specific *let-7* family members in at least these three types of human cancers. However, this mechanism is likely cancer-type specific, since we did not find significant copy number alterations of the *let-7* family in other cancer types, such as colon and prostate cancers.

Importantly, focal reductions in the copy numbers of the *let-7* family suggest that deletion of *let-7* may play an important role during tumorigenesis, and suggests that restoring expression of these *let-7* family members may be a novel strategy to treat medulloblastoma, breast cancer, and ovarian cancer. Current rapid advances in oligonucleotide/nanoparticle therapy create realistic optimism for the establishment of miRNAs as a new and potent therapeutic target and/or chemoresistant modulator in cancer. To test this hypothesis, we delivered a small RNA mimic for *let-7b*, the most frequently deleted *let-7* family member in ovarian cancer patients, to ovarian cancer cells *in vitro* and *in vivo*. We found that restoration of *let-7b* expression dramatically inhibited tumor growth. In agreement with our observations, restoration of *let-7* expression has also been shown to reduce tumor growth in pre-clinical models of other cancer types, such as lung cancer [13]–[16], in which the *let-7* family is globally decreased [9], [10]. Given that *let-7* simultaneously inhibits multiple oncogenic pathways that are involved in most steps of tumorigenesis (such as *RAS, MYC*, and *HMGA2)*, restoration of *let-7* expression in tumor cells provides a novel therapeutic strategy to treat cancer. Interestingly, we found that our *let-7b* treatment did not significantly affect normal ovarian surface epithelial cell growth, suggesting that treatment to restore *let-7* expression may be less toxic than traditional chemotherapy. This finding may be due to the fact that normal cells already express higher levels of endogenous *let-7* and therefore the delivery of additional *let-7* does not significantly increase its gene silencing activity in normal cells. Another explanation could be that the downstream cellular context is different in the tumor cells vs. the normal cells. For example, some direct targets of *let-7,* such as *LIN28, RAS, MYC* and *HMGA2,* are not expressed or activated in normal cells, but are the ‘driver’ genes promoting cell growth in tumors.

It has been demonstrated by independent laboratories that the repression on their target gene expression levels by miRNAs is relatively mild [56]–[59], although each miRNA can specifically and simultaneously target hundreds of mRNA transcripts [56], [59]. In consistent with this common behavior of miRNAs, we showed that the mRNA expression levels of multiple well-known *let-7* target genes such as *CCND1*, *CDC25A*, *HMGA2*, *IL6* and *LIN28B* were significantly decreased by *let-7b* mimic treatment (all p<0.05). However, the repression effects on the mRNA levels by *let-7b* are typically modest (from 58.0% ±0.03% to 78.9% ±0.08%). It could bring important therapeutic benefits for the *let-7b* replacement therapy compared to the siRNA based strategies: First, the side effects of restoration of *let-7b* in patients with cancer may be mild and limited. Second, multiple oncogenic genes/pathways such as cell cycle regulation, DNA damage response, cancer stem cell differentiation and inflammation could be targeted simultaneously. In agree with this hypothesis, indeed we did not find that the *let-7b* replacement therapy significantly affected on normal epithelial cell growth at a therapeutic dosage (Figure 4C). In addition, miRNAs including *let-7* negatively regulate target gene expression by two major mechanisms, i.e. mRNA cleavage (transcriptional level) and/or translational repression (translational level), in a sequence-specific manner [7], [8], [11], [12]. This could be another explanation why we only found modest repression of the target gene expression in mRNA levels that were detected by real-time RT-PCR. Finally, the dosage for *let-7b* delivery in the present study was determined by a dose-dependent experiment. We used the minimal functional response dosage (30 nM) for our real-time RT-PCR experiments. When we increased the dose of *let-7b* delivery, we found the effects of the *let-7b* replacement therapy on its target genes were remarkably increased.

It has been well demonstrated that the mature *let-7* expression is a robust biomarker to predict clinical outcome in patients with cancer. For example, a systematic review of 43 published studies shows that *let-7* is the miRNA most frequently and significantly associated with clinical outcomes in patients with cancer [60]. To examine the correlation between the *let-7b/let-7a3* cluster copy number alterations (deletion v.s. non-deletion) and clinical outcome of patients, a total of 491 late-stage (stage-III and -IV) tumors with well-annotated survival information was examined by Kaplan-Meier survival analysis. However, we did not find significant correlation between the *let-7b/let-7a3* cluster copy number alterations and overall survival in this sample set (p = 0.343). This result indicated that the DNA copy number alteration is not the only reason by which the mature *let-7* expression is reduced in cancer. In agree with this observation, we and other independent groups have reported that miRNA deregulation in human ovarian cancer was led by multiple mechanisms [29], [61], [62]. First, it has been shown that most primary miRNAs are transcripted from Pol II promoter and regulated by transcriptional factors. Several examples showing miRNA deregulation in ovarian cancer by transcriptional deregulation have been reported. Second, recent studies suggest that epigenetic alterations play a critical role in deregulating miRNA expression in human ovarian cancers. Third, mutation might also contribute to down-regulation of mature miRNAs. Finally, the key proteins in the miRNA biogenesis pathway may be dysfunctional or deregulated in cancer, and may enhance tumorigenesis. Therefore, transcriptional deregulations, epigenetic alterations, mutations, DNA copy number abnormalities and defects in the miRNA biogenesis machinery might each contribute, either alone but more likely together, to the *let-7* family deregulation in human cancer [29], [61], [62]. In the present study, we reported that *let-7b/let-7a3* cluster deleted in more than 40% percentage of ovarian and breast cancers. We believe it is one of important mechanisms to decrease *let-7* expression in these diseases, although other mechanisms such as transcriptional deregulations, epigenetic alterations, mutations, and defects in the miRNA biogenesis machinery are needed to be further characterized in ovarian and breast cancers.

## Materials and Methods

### SNP and miRNA Microarray Data Retrieval and Analysis

The segmental raw data from the SNP microarrays (Affymetrix 250 K Sty array) was downloaded from the Tumorscape database (created by the Broad Institute of MIT and Harvard) [27] and analyzed following a standard protocol provided by Tumorscape, then visualized using the Integrative Genomics Viewer (IGV) (Figure 1) [30]. Fourteen types of human cancers (a total of 2,969 tumor specimens) were included in our study (Table 1). The level 3 data (segmented SNP array data and normalized miRNA array data) was download from The Cancer Genome Atlas (TCGA) dataset [32], and a total of 537 tumors with both SNP and miRNA array data were identified. The genomic regions where copy number alterations occurred significantly more frequently than the background rate were identified using the Genomic Identification of Significant Targets in Cancer (GISTIC) algorithm [31]. The information for the tumor types of the specimens from the Tumorscape database was available at the portal of the Tumorscape (http://www.broadinstitute.org/tumorscape) [27]. Breast and ovarian tumors clinical information was available from the published profiles (GSE7545 and GSE19399) [27], [63], [64]. All TCGA ovarian cancer specimens are high-grade serous ovarian tumors, which were collected from newly diagnosed patients with ovarian serous adenocarcinoma who were undergoing surgical resection and had received no prior treatment for their disease, including chemotherapy or radiotherapy. All cases had to be of serous histology but were collected regardless of surgical stage or histologic grade. Cases were staged according to the 1988 FIGO staging system. The detail clinical information of the specimens from the TCGA database was available at the TCGA data portal (https://tcga-data.nci.nih.gov/tcga/) [32].

### Cell Lines and Cell Culture

Ovarian cancer cell lines were purchased from the American Type Culture Collection (ATCC) and the Division of Cancer Treatment and Diagnosis (DCTD) Tumor/Cell Line Repository. All cancer cell lines were cultured in RPMI 1640 medium (Invitrogen) supplemented with 10% fetal bovine serum (Invitrogen). Human ovarian surface epithelial (HOSE) cells were generously provided by Dr. Nelly Auersperg (University of British Columbia) [65]. HOSE cells were cultured 1∶1 in Media 199: MCDB 105 (Sigma), supplemented with 15% FBS.

### *let-7b* Mimic *in vitro* Transfection

The *let-7b* mimic and control oligonucleotides were purchased from Invitrogen. Cells were plated 24 hours before transfection. Transfection of the miRNA oligonucleotides (30 nM) *in vitro* was performed using Lipofectamine RNAiMAX (Invitrogen).

### MTT Assay

MTT assays were performed in 96-well plates using a Cell Proliferation Kit (Roche) following the manufacturer’s instructions. Four to six wells were analyzed for each sample. The resulting colored solution was quantified using an ELx800 Absorbance Microplate Reader (BioTek) at 570 nm with a reference wavelength of 630 nm.

### Generation of Stable Cell Lines

The let-7 reporter vector was transfected into A2780 cells using the FuGene6 Transfection Reagent (Roche). The medium was changed 48 hrs post-transfection, and the stable clones were selected by neomycin for 14 days. The stable clones expressing the let-7 reporter were further confirmed by a luciferase assay.

### Generation of Orthotopic Ovarian Cancer Xenograft Model

Six to eight week old female nude mice were obtained from The Jackson Laboratory. Subconfluent ovarian cancer cells (A2780) were trypsinized and suspended in phosphate buffered saline (PBS), then 10×10^6^ cells in a total volume of 0.1 ml were injected into the mouse peritoneal cavity. The animal study protocol was reviewed and approved by the institutional animal care and use committee (IACUC) of the University of Pennsylvania.

### *let-7b* Mimic Delivery *in vivo*

The *let-7b* mimic and control oligonucleotides were chemically synthesized by Sigma, and the jetPEI reagent (Polyplus Transfection) was used to deliver the miRNA *in vivo*. Two weeks after the tumor cell injection (Figure 5A), the mice were randomly assigned to two groups, to be treated with either the *let-7* mimic or the control oligonucleotide (40 ug per animal). The *let-7b* mimic (or the control oligonucleotide) was combined with the jetPEI reagent at an N/P ratio of 8, following the manufacturer’s instructions. The *let7*-mimic/jetPEI or control-mimic/jetPEI was administrated to the mice by intraperitoneal injection every three days for a total of four treatments (Figure 5A). Three days after the last treatment, the animals were euthanized and the tumor nodes were collected and weighed.

### Bioluminescence Optical Imaging

The IVIS Lumina II Bioluminescence and Fluorescence Imaging System (Caliper Life Sciences) was used for *in vivo* bioluminescent imaging of the xenograft tumors. Animals were anesthetized with isoflurane and injected intraperitoneally with D-luciferin substrate (150 mg/kg body weight) (Caliper Life Sciences). Pilot studies revealed that the peak bioluminescent intensity of the tumors was reached 10 minutes after D-luciferin injection; therefore, this time point was chosen for imaging. Images of the tumor were taken under the following settings: exposure time = 0.5 seconds, f/stop = 16, medium binning. Living Image software was used to quantify the bioluminescent signal, reported as units of tissue radiance (photons/s/cm2/sr).

### RNA Isolation

Total RNA was isolated from 100–500 mg of frozen tissue using TRIzol reagent (Invitrogen). The quality and quantity of the isolated RNA was analyzed using a Bioanalyzer 2100 system (Agilent).

### TaqMan miRNA Assays

Expression of mature *let-7b* was analyzed using TaqMan miRNA assays (Applied Biosystems, Foster City, CA, USA) under conditions recommended by the manufacture. Briefly, single-stranded cDNA was synthesized from 5 ng of total RNA in a 15 µl reaction volume using a TaqMan microRNA reverse transcription kit (Applied Biosystems, Foster City, CA, USA). The reactions were incubated first at 16°C for 30 min, at 42°C for 30 min, and then inactivated by incubation at 85°C for 5 minutes. Each cDNA generated was amplified by quantitative PCR using sequence-specific primers from the TaqMan microRNA assay on an 7900HT sequence detection system (Applied Biosystems, Foster City, CA, USA). Each 20 µl PCR included 10 µl of 2× Universal PCR Master Mix (without AmpErase UNG), 1 µl of 20× TaqMan microRNA assay mix, and 2 µl of reverse transcription product. The reactions were incubated in a 384-well plate at 95°C for 10 min, followed by 40 cycles of 95°C for 15 seconds and 60°C for 1 minute.

### Quantitative QRT-PCR

Total RNA was reverse-transcribed using a High Capacity RNA-to-cDNA Kit (Applied Biosystems) according to the manufacturer’s instructions. cDNA was quantified by real-time PCR on an ABI Prism 7900 Sequence Detection System (Applied Biosystems). PCR was performed using SYBR Green PCR Core reagents (Applied Biosystems) according to manufacturer’s instructions. PCR amplification of the housekeeping gene GAPDH was performed for each sample as a control for sample loading and to allow for normalization across samples.

### Statistical Analysis

Statistical analysis was performed using the SPSS and SAS statistical software packages.
